# Exploring the intersectionality of race/ethnicity with rurality on breast cancer outcomes: SEER analysis, 2000–2016

**DOI:** 10.1007/s10549-022-06830-x

**Published:** 2022-12-15

**Authors:** Justin Xavier Moore, Sydney Elizabeth Andrzejak, Samantha Jones, Yunan Han

**Affiliations:** 1 Cancer Prevention, Control, & Population Health, Medical College of Georgia, Georgia Cancer Center, Augusta University, Augusta, GA USA; 2 Institute of Preventive and Public Health, Medical College of Georgia, Augusta University, Augusta, GA USA; 3 Division of Public Health Sciences, Department of Surgery, Washington University School of Medicine, St. Louis, MO 63110 USA; 4 Cancer Prevention, Control, & Population Health Program, Department of Medicine, Institute of Public and Preventive Health, Medical College of Georgia at Augusta University, 1410 Laney Walker Blvd. CN-2135, Augusta, GA 30912 USA

**Keywords:** Cancer, Breast cancer, Rural/urban, Race, Disparities

## Abstract

**Purpose:**

Disparities in breast cancer survival have been observed within marginalized racial/ethnic groups and within the rural–urban continuum for decades. We examined whether there were differences among the intersectionality of race/ethnicity and rural residence on breast cancer outcomes.

**Methods:**

We performed a retrospective analysis among 739,448 breast cancer patients using Surveillance Epidemiology and End Results (SEER) 18 registries years 2000 through 2016. We conducted multilevel logistic-regression and Cox proportional hazards models to estimate adjusted odds ratios (AORs) and hazard ratios (AHRs), respectively, for breast cancer outcomes including surgical treatment, radiation therapy, chemotherapy, late-stage disease, and risk of breast cancer death. Rural was defined as 2013 Rural–Urban Continuum Codes (RUCC) of 4 or greater.

**Results:**

Compared with non-Hispanic white–urban (NH-white–U) women, NH-black–U, NH-black–rural (R), Hispanic–U, and Hispanic–R women, respectively, were at increased odds of no receipt of surgical treatment (NH-black–U, AOR = 1.98, 95% CI 1.91–2.05; NH-black–R, AOR = 1.72, 95% CI 1.52–1.94; Hispanic–U, AOR = 1.58, 95% CI 1.52–1.65; and Hispanic–R, AOR = 1.40, 95% CI 1.18–1.67), late-stage diagnosis (NH-black–U, AOR = 1.32, 95% CI 1.29–1.34; NH-black–R, AOR = 1.29, 95% CI 1.22–1.36; Hispanic–U, AOR = 1.25, 95% CI 1.23–1.27; and Hispanic–R, AOR = 1.17, 95% CI 1.08–1.27), and increased risks for breast cancer death (NH-black–U, AHR = 1.46, 95% CI 1.43–1.50; NH-black–R, AHR = 1.42, 95% CI 1.32–1.53; and Hispanic–U, AHR = 1.10, 95% CI 1.07–1.13).

**Conclusion:**

Regardless of rurality, NH-black and Hispanic women had significantly increased odds of late-stage diagnosis, no receipt of treatment, and risk of breast cancer death.

**Supplementary Information:**

The online version contains supplementary material available at 10.1007/s10549-022-06830-x.

## Introduction

Decades of research shed light to the inequitable access to healthcare across geographic locations. Rural populations have a greater prevalence rate of chronic diseases, higher incidence of late-stage cancers, and higher mortality rate than urban populations [1–3]. Nationally, incidence and mortality rates of lung, colorectal, and particularly breast cancer have decreased in recent years, although this progress has not equally affected rural, lower socioeconomic, or racial/ethnic minority populations [4–10]. In 2021, Non-Hispanic white (NH-white) women had a highest incidence of breast cancer (131.8 per 100,000), followed by Non-Hispanic black (NH-black) women (124.7 per 100,000), Asian/Pacific Islander (API) women (105.1 per 100,000), and Hispanic women (100.3 per 100,000) [11]. Despite this, NH-black women have disproportionately higher breast cancer mortality rates compared to other race-ethnicities, particularly NH-white women (27.1 deaths per 100,000 for NH-black women vs 19.4 deaths per 100,000 for NH-white women) [11].

These disparities are partly explained by social determinants of health, including socioeconomic inequalities [12, 13], differential access to high-quality screening and healthcare resources [14, 15], factors that influence stage at diagnosis and receipt of guideline-adherent treatment [16], and therefore survival [17]. Moreover, molecular subtypes of breast cancer have been shown to have varying prevalence by race. Particularly, premenopausal Hispanic and NH-black women are more likely to be diagnosed with triple-negative breast cancer compared to women of other race/ethnicities [18, 19].

Understanding the influence geographic location has on accessibility to healthcare can bridge gaps in rural–urban disparities. Those living in rural communities often experience additional barriers to appropriate care across the cancer continuum, including inadequate screening [20–22], prolonged follow-up after abnormal screening tests [23, 24], and excess travel time and distance for treatment of diagnosed cancers [16, 25, 26]. A recent study by *Mobley *et al*. (2021)* reported that counties in the United States (U.S.) with persistent “hot spots” for breast cancer late-stage diagnosis were located within deprived areas characterized by lower socioeconomic status SES, lower levels of health insurance, decreased access to mammography screening, and more isolated minority (racially segregated) neighborhoods [27]. Moreover, another study using the Georgia cancer registry observed a 53% increased risk of breast cancer death among NH-black rural women, when compared to NH-white rural women [28]. Similarly, *Singh *et al*. (2011)* observed that there was a 6.2% increase in breast cancer mortality for every unit increase in rural–urban continuum (i.e., more rural counties) among all women, a 7.9 unit increase among NH-white women, and a 1.3% unit increase among NH-black women, though non-significant [29]. In this study, we sought to examine whether there were differences on the intersectionality of race/ethnicity and rurality with breast cancer outcomes.

## Methods

### Data source

We utilized data from the Surveillance Epidemiology and End Results (SEER) 18 registries custom data (with additional treatment fields) (http://seer.cancer.gov), November 2018 submission (1975–2016 varying) linked with county-level attributes. Since 1973, the SEER program provides information regarding cancer statistics in effort to reduce the cancer burden among the U.S. population and the data are collected and curated by the National Cancer Institute Division of Cancer Control and Population Sciences. Specifically, SEER 18 registries cover approximately 27.8% of the U.S. population (based on the 2010 Census) including cancer patient data from 18 geographic areas and cancer registries [30].

### Ethical statement

This study was considered exempt by the Institutional Review Boards of Augusta University and Washington University School of Medicine because we utilized pre-existing secondary data that are publicly available and deidentified.

### Study population

We performed the “case listing” function through SEER*Stat software (version 8.3.6) to export potential cases, a total of 1,187,514 breast cancer patients diagnosed between 1975 and 2016, for analysis from the SEER 18 database. Women were excluded if they were diagnosed prior to 2000 (*n* = 348,245); had unknown rural or urban designation (*n* = 1148); were missing follow-up time (*n* = 4282); were missing information breast cancer stage at diagnosis (*n* = 67,471); had unknown age (*n* = 33); had other or unknown race/ethnicity (*n* = 6428), and breast cancer was not first primary cancer diagnosis (*n* = 20,459). Corresponding, our analytic sample consisted of 739,448 female breast cancer women for analysis.

### Exposure(s) of interest and potential confounders

Our primary exposures of interest were race/ethnicity and rural–urban designation for each patient. SEER coded race and ethnicity data were obtained through electronic medical records, provider notes, photographs, and any other sources used to determine race [31]. Race/ethnicity categories included non-Hispanic white, non-Hispanic black, non-Hispanic Asian/Pacific Islander, and Hispanic. To classify the urban or rural women, we utilized the 2013 Rural–Urban Continuum Codes (RUCC) [32]. The 2013 RUCCs classify metropolitan counties by population size and non-metropolitan counties by the degree of urbanization and their proximity to a metropolitan area. Consistent with previous urban–rural thresholds [33–35], we classified women in counties with codes 1–3 as urban, while counties coded 4–9 were categorized as rural. We subsequently categorized women based on their race/ethnicity and rural–urban status into eight mutually exclusive groups: (1) NH-white/urban, (2) NH-black/urban, (3) API/urban, (4) Hispanic/urban, (5) NH-white/rural, (6) NH-black/rural, (7) API/rural, and (8) Hispanic/rural. Potential confounders that were known risk factors for breast cancer survival included patient-level characteristics (e.g., age, marital status), and tumor characteristics (e.g., breast cancer subtype, laterality). The classification of cancer status is based on SEER variables relating to the hormone receptor status of tumors recorded by the SEER program [36]. SEER provided estrogen receptor (ER) and progesterone receptor (PR) status of breast tumors since 1990, but not for human epidermal growth factor receptor (HER2) until data collected 2010 and later. Thus, we only reported ER/PR status for breast cancer status for consistency over study period.

We included county-level attributes [37] pertaining to socioeconomic/demographic (SES) and health care access (HCA). SEER investigators linked each patient (case-listing) with county-level attributes obtained from the Census’ American Community Survey (ACS) 5-year summary files based on the county of their residence at cancer diagnosis. We selected 2013–2017 ACS county attributes for this analysis to provide the area-level measures that were consistent with the latest observed period of incident cases. SES county attributes that were included are percentage of county population with less than 9th grade education, percentage of families living with household income below the federal poverty level, and the percentage of county population ages 16 and older who are unemployed. HCA county attributes that were included are percentage of women aged 40 and older with a mammography screening with prior 2 years small-area estimation from 2008 to 2010, ratio of population to primary care physicians (adapted from the 2017 Area Health Resource File/American Medical Association via the 2020 County Health Ranking’s file), and percentage of county population uninsured (adapted from the 2017 Small Area Health Insurance Estimates via the 2020 County Health Ranking’s file) [38].

### Outcome(s) of interest

There were five outcome variables of interest in this study: (1) stage at diagnosis based on the SEER summary stage variable and categorized as late stage (if regional or distant) or early stage (if in situ and localized); (2) surgical treatment given or not, regardless of reason; (3) radiation treatment given or not, regardless of reason; (4) chemotherapy treatment given or not, regardless of reason; and (5) breast cancer-specific death and time to death [39].

### Statistical analysis

We compared the distribution of the SEER sample characteristics between groups of race/ethnicity and rural–urban status using Chi-square tests for categorical variables. We presented these descriptive statistics as the count and relative frequencies (percentages) for each categorical variable. We conducted consecutive multilevel logistic-regression (generalized linear mixed models) models for the binary outcomes of (1) late-stage diagnosis, (2) no surgical treatment, (3) no radiation therapy, and (4) no chemotherapy. The estimates derived from these multilevel logistic-regression models are interpreted as adjusted odds ratios (AORs) and 95% confidence intervals (CIs). We examined the proportional hazards assumption for breast cancer-specific mortality by Schoenfeld residuals, and by graphically assessing the log–log plots of survival. After confirming the proportionality of hazards assumption, we estimated the hazard/risk of breast cancer death by each group of race/ethnicity and rural–urban status (referent group was NH-white/urban women) and fit Cox proportional hazards models with time-to-breast cancer-related death as the outcome and censored women at the time of death, or the end of follow-up (December 31, 2016). The mean follow-up time was 6.67 years (standard deviation = 4.69). We estimated the mean survival times using the product-limit method of the Kaplan–Meier survival estimator. We examined the survival function of cancer mortality by rural/urban status overall, and then stratified by race/ethnicity using the Kaplan–Meier method. The estimates derived from the Cox proportional hazards models are interpreted as adjusted hazard ratios (AHRs) and associated 95% CIs. We performed four adjusted models to understand the effect of the intersectionality of race/ethnicity with rurality on breast cancer outcomes by accounting for known potential confounders considering the social determinants of health (SES and HCA): (1) the first adjusted for age, SEER registry, and ER/PR status; (2) SES adjusted model which included age, SEER registry, and county SES attributes of county population with less than 9th grade education, percentage of families living with household income below the federal poverty level, and the percentage of county population aged 16 and older who are unemployed; (3) HCA adjusted model which included age, SEER registry, and county HCA attributes of percentage of women aged 40 and older with a mammography screening with prior 2 years small-area estimation from 2008 to 2010, ratio of population to primary care physicians, and percentage of county population uninsured; and (4) a fully adjusted model accounting for age, SEER registry, HR status, and both county SES and HCA attributes, late-stage diagnosis, surgery, radiation, and chemotherapy treatment. In secondary analyses, we performed fully adjusted models and stratified by breast cancer estrogen receptor (ER)/ progesterone receptor (PR) status. We used SAS version 9.4 for all statistical analyses. We considered *p* values ≤ 0.05 and confidence intervals excluding the null value (odds ratio = 1.00) as statistically significant.

## Results

### Sociodemographic of SEER women 2000—2016

Table 1 displays demographics of SEER participants (*n* = 739,448) diagnosed with breast cancer between 2000 and 2016. The average age of participants was 60.2 years, and 56% of SEER participants were married or had a domestic partner. Among the breast cancer patients, NH-black–rural (40.9%), urban (40.2%) and Hispanic–urban (39.2%), and rural (36.3%) women were more likely to be diagnosed with late-stage breast cancer compared to their NH-white counterparts (*p* value < 0.001). NH-black women in urban (44.2%) and rural (43.6%) locations are more likely to be diagnosed with a grade III tumor, when all other women were more likely to be diagnosed with a grade II tumor (NH-white 41.6% urban vs 40.3% rural; API 41.5% urban vs 45.9% rural; Hispanic 39.0% urban vs 38.4% rural). NH-black urban (27.6%), rural (27.2%) and Hispanic–urban (18.9%) and rural women (17.4%) were more likely to be diagnosed with ER-/PR- tumor status compared to their NH-white counterparts (*p* value < 0.001). NH-black urban (6.7%), rural (6.0%), and Hispanic–rural women (6.1%) are more likely to not undergo surgical treatment, compared to NH-white women (*p* value < 0.001). NH-white–urban women were more likely to have undergone radiation (53.0%), when all other participants were more likely to not receive radiation treatment. However, Hispanic–urban women, NH-black–urban women, and NH-black–rural women were more likely to undergo chemotherapy, 48.5%, 50.8%, and 50.4% respectively.

**Table 1:** Summary of population characteristics of 739,448 participants by race/ethnicity and rural residence, among Surveillance Epidemiology and End Results (SEER) women diagnosed between 2000 and 2016

	Urban (column %)				Rural (column %)				
	NH–white (*n* = 463,627)	NH–black (*n* = 70,282)	API (*n* = 57,191)	Hispanic (*n* = 74,345)	NH–white (*n* = 64,012)	NH–black (*n* = 5,645)	API (*n* = 1,650)	Hispanic (*n* = 2,696)	Overall (739,448)
Characteristics									
Age									
< 40	20,382 (4.4)	5694 (8.1)	4564 (8.0)	7447 (10.0)	2404 (3.8)	386 (6.8)	118 (7.2)	241 (8.9)	41,236
40–49	76,365 (16.5)	15,060 (21.4)	14,061 (24.6)	19,038 (25.6)	8882 (13.9)	1130 (20.0)	277 (16.8)	560 (20.8)	135,373
50–59	113,789 (24.5)	19,155 (27.3)	15,533 (27.2)	19,726 (26.5)	14,855 (23.2)	1493 (26.5)	406 (24.6)	677 (25.1)	185,634
60–69	116,224 (25.1)	15,804 (22.5)	12,776 (22.3)	15,331 (20.6)	17,247 (26.9)	1303 (23.1)	450 (27.3)	658 (24.4)	179,793
70 +	136,867 (29.5)	14,569 (20.7)	10,257 (17.9)	12,803 (17.2)	20,624 (32.2)	1333 (23.6)	399 (24.2)	560 (20.8)	197,412
Marital status at diagnosis									
Single	52,401 (11.3)	20,218 (28.8)	7138 (12.5)	13,420 (18.1)	4667 (7.3)	1348 (23.9)	204 (12.4)	354 (13.1)	99,750
Married/domestic partner	268,885 (58.0)	24,970 (35.5)	37,862 (66.2)	41,084 (55.3)	38,005 (59.4)	2069 (36.7)	983 (59.6)	1435 (53.2)	415,293
Divorced/separated/widow	123,030 (26.5)	21,744 (30.9)	10,236 (17.9)	16,364 (22.0)	18,148 (28.4)	1882 (33.3)	361 (21.9)	583 (21.6)	192,348
Unknown	19,311 (4.2)	3350 (4.8)	1955 (3.4)	3477 (4.7)	3192 (5.0)	346 (6.1)	102 (6.2)	324 (12.0)	32,057
Late-stage diagnosis^a^									
No	317,963 (68.6)	42,023 (59.8)	38,401 (67.2)	45,201 (60.8)	43,790 (68.4)	3336 (59.1)	1164 (70.6)	1718 (63.7)	493,596
Yes	145,664 (31.4)	28,259 (40.2)	18,790 (32.8)	29,144 (39.2)	20,222 (31.6)	2309 (40.9)	486 (29.5)	978 (36.3)	245,852
Tumor stage									
Localized	317,963 (68.6)	42,023 (59.8)	38,401 (67.2)	45,201 (60.8)	43,790 (68.4)	3336 (59.1)	1164 (70.6)	1718 (63.7)	493,596
Regionalized	143,933 (31.0)	27,813 (39.6)	18,534 (32.4)	28,779 (38.7)	19,954 (31.2)	2275 (40.3)	483 (29.3)	968 (35.9)	242,739
Distant	1731 (0.4)	446 (0.6)	256 (0.5)	365 (0.5)	268 (0.4)	34 (0.6)	3 (0.2)	10 (0.4)	3113
Tumor grade									
Grade I	107,030 (23.1)	9361 (13.3)	10,743 (18.8)	12,812 (17.2)	14,041 (21.9)	796 (14.1)	311 (18.9 )	533 (19.8)	155,627
Grade II	192,723 (41.6)	9361 (13.3)	23,741 (41.5)	29,014 (39.0)	25,820 (40.3)	1810 (32.1)	757 (45.9)	1036 (38.4)	298,957
Grade III	131,581 (28.4)	31,042 (44.2)	18,881 (33.0)	27,066 (36.4)	18,993 (29.7)	2459 (43.6)	496 (30.1)	903 (33.5)	231,421
Grade IV	3713 (0.8)	778 (1.1)	598 (1.1)	912 (1.2)	761 (1.2)	87 (1.5)	25 (1.5)	28 (1.0)	6902
Unknown	28,580 (6.2)	5045 (7.2)	3228 (5.6)	4541 (6.1)	4397 (6.9)	493 (8.7)	61 (3.7)	196 (7.3)	46,541
Surgery									
Yes	446,936 (96.4)	65,586 (93.3)	54,911 (96.0)	70,537 (94.9)	61,716 (96.4)	5,304 (94.0)	1612 (97.7)	2531 (93.9)	709,135
No/unknown	16,689 (3.6)	4696 (6.7)	2280 (4.0)	3808 (5.1)	2296 (3.6)	341 (6.0)	38 (2.3)	165 (6.1)	30,313
Radiation									
Yes	245,705 (53.0)	34,835 (49.6)	27,813 (48.6)	34,511 (46.4)	29,964 (46.8)	2493 (44.2)	745 (45.2)	1,207 (44.8)	377,273
No/unknown	217,922 (47.0)	35,447 (50.4)	29,378 (51.4)	39,834 (53.6)	34,048 (53.2)	3152 (55.8)	905 (54.8)	1,489 (55.2)	362,175
Chemotherapy									
Yes	173,021 (37.3)	35,729 (50.8)	25,321 (44.3)	36,051 (48.5)	24,468 (38.2)	2845 (50.4)	653 (39.6)	1270 (47.1)	299,358
No/unknown	290,606 (62.7)	34,553 (49.2)	31,870 (55.7)	38,294 (51.5)	39,544 (61.8)	2800 (49.6)	997 (60.4)	1426 (52.9)	440,090
Status									
ER+/PR+	305,786 (65.9)	35,427 (50.4)	37,362 (65.3)	44,960 (60.5)	40,817 (63.8)	2702 (47.9)	1104 (66.9)	1629 (60.4)	469,787
ER+/PR– or ER–/PR+	60,511 (13.1)	10,326 (14.7)	7440 (13.0)	9831 (13.2)	7746 (12.1)	764 (13.5)	206 (12.5)	311 (11.5)	97,135
ER-/PR-	66,478 (14.3)	19,396 (27.6)	9273 (16.2)	14,080 (18.9)	10,140 (15.8)	1537 (27.2)	249 (15.1)	468 (17.4)	121,621
Unknown	30,852 (6.7)	5,133 (7.3)	3116 (5,5)	5474 (7.4)	5,309 (8.3)	642 (11.4)	91 (5.5)	288 (10.7)	50,905
Breast cancer laterality									
Right	229,163 (49.4)	34,329 (48.8)	28,202 (49.3)	36,304 (48.8)	31,533 (49.3)	2720 (48.2)	834 (50.6)	1300 (48.2)	364,385
Left	234,085 (50.5)	35,884 (51.1)	28,954 (50.6)	37,985 (51.1)	32,430 (50.7)	2916 (51.7)	816 (49.5)	1,394 (51.7)	374,464
Bilateral	30 (0.01)	13 (0.02)	4 (0.01)	7 (0.01)	6 (0.01)	0 (0.0)	0 (0.0)	0 (0.0)	60
Unknown	349 (0.08)	56 (0.08)	31 (0.05)	49 (0.07)	43 (0.07)	9 (0.1)	0 (0.0)	2 (0.1)	539

^a^Yes = Distant and Regionalized

### Odds for no surgical treatment

We performed several multivariable models to assess the possible influence of specific confounders on the relationship between race/ethnicity and rurality on breast cancer outcomes (Table 2). When adjusted for ER/PR status alone, NH-black–urban women (AOR: 1.97, 95% CI 1.90–2.04), NH-black–rural women (AOR: 1.75, 95% CI 1.55–1.96), Hispanic–urban women (AOR: 1.58, 95% CI 1.52–1.64), Hispanic–rural women (AOR: 1.39, 95% CI 1.18–1.65), and API–urban women (AOR: 1.36, 95% CI 1.30–1.43) had an increased odds of no surgical treatment, when compared to NH-white–urban women. However, when only accounting for county-level SES NH-black–urban women (AOR: 2.07, 95% CI: 2.00 – 2.15), NH-black–rural women (AOR: 1.86, 95% CI 1.65–2.09), Hispanic–urban women (AOR: 1.59, 95% CI: 1.53 – 1.66), Hispanic–rural women (AOR: 1.43, 95% CI 1.21–1.69), API–urban women (AOR: 1.36, 95% CI 1.29–1.42), and NH-white–rural women (AOR: 1.08, 95% CI 1.03–1.14) had an increased odds of no surgical treatment, when compared to NH-white–urban women. Results in fully adjusted models reflected similar trends as ER/PR only adjusted models.

**Table 2:** Multivariable Adjusted Odds Ratios for No Surgical Treatment, No Chemotherapy, and No Radiation Therapy for SEER Breast Cancer women diagnosed between 2000 and 2016

	ER/PR status AOR (95% CI)^**a**^	SES AOR (95% CI)^b^	HCA AOR (95% CI)^c^	Fully adjusted AOR (95% CI)^**d**^
Odds for no surgical treatment				
Race/Ethnicity-rurality				
NH-white–Urban (Referent)	1.00	1.00	1.00	1.00
NH-black–Urban	1.97 (1.90–2.04)	2.07 (2.00–2.15)	2.04 (1.97–2.12)	1.98 (1.91–2.05)
API–Urban	1.36 (1.30–1.43)	1.36 (1.29–1.42)	1.35 (1.29–1.42)	1.36 (1.29–1.42)
Hispanic–Urban	1.58 (1.52–1.64)	1.59 (1.53–1.66)	1.59 (1.53–1.65)	1.58 (1.52–1.65)
NH-white–Rural	1.03 (0.98–1.09)	1.08 (1.03–1.14)	1.11 (1.06–1.17)	1.05 (1.00–1.11)
NH-black–Rural	1.75 (1.55–1.96)	1.86 (1.65–2.09)	1.92 (1.71–2.16)	1.72 (1.52–1.94)
API–Rural	0.90 (0.64–1.25)	0.95 (0.68–1.32)	1.02 (0.73–1.43)	0.96 (0.68–1.34)
Hispanic–Rural	1.39 (1.18–1.65)	1.43 (1.21–1.69)	1.47(1.24–1.75)	1.40 (1.18–1.67)
Odds for no radiation therapy				
Race/Ethnicity-rurality				
NH-white–Urban (Referent)	1.00	1.00	1.00	1.00
NH-black–Urban	1.16 (1.14–1.18)	1.18 (1.16–1.20)	1.19 (1.17–1.21)	1.14 (1.12–1.16)
API–Urban	1.33 (1.30–1.36)	1.32 (1.20–1.35)	1.33 (1.30–1.35)	1.33 (1.30–1.35)
Hispanic–Urban	1.21 (1.19–1.23)	1.20 (1.18–1.22)	1.21 (1.19–1.23)	1.19 (1.17–1.21)
NH-white–Rural	1.27 (1.24–1.29)	1.24 (1.21–1.26)	1.25 (1.23–1.28)	1.21 (1.19–1.23)
NH-black–Rural	1.31 (1.24–1.38)	1.25 (1.18–1.32)	1.33 (1.26–1.41)	1.18 (1.12–1.25)
API–Rural	2.16 (1.94–2.39)	2.09 (1.89–2.32)	2.14 (1.93–2.37)	2.08 (1.87–2.31)
Hispanic–Rural	1.31 (1.21–1.41)	1.27 (1.17–1.38)	1.29 (1.19–1.40)	1.25 (1.15–1.35)
Odds for no chemotherapy				
Race/Ethnicity-rurality				
NH-white–Urban (Referent)	1.00	1.00	1.00	1.00
NH-black–Urban	0.82 (0.81–0.84)	0.70 (0.69–0.72)	0.69 (0.68–0.70)	0.83 (0.81–0.85)
API–Urban	0.95 (0.93–0.97)	0.93 (0.91–0.95)	0.93 (0.91–0.94)	0.95 (0.93–0.97)
Hispanic–Urban	0.83 (0.81–0.85)	0.81 (0.79–0.82)	0.80 (0.78–0.81)	0.84 (0.82–0.86)
NH-white–Rural	0.93 (0.92–0.95)	0.97 (0.95–0.99)	0.97 (0.95–0.99)	0.97 (0.95–0.99)
NH-black–Rural	0.82 (0.77–0.87)	0.75 (0.71–0.80)	0.72 (0.68–0.77)	0.86 (0.81–0.92)
API–Rural	0.98 (0.87–1.10)	1.00 (0.89–1.12)	1.04 (0.92–1.16)	1.02 (0.91–1.15)
Hispanic–Rural	0.78 (0.71–0.85)	0.79 (0.72–0.82)	0.80 (0.73–0.87)	0.81 (0.74–0.89)

^a^Adjusted for age, SEER registry, and ER/PR status

^b^Adjusted for age, SEER registry, and county-level SES

^c^Adjusted for age, SEER registry, and county-level HCA

^d^Adjusted for age, SEER registry, ER/PR status, county-level SES, and county-level HCA

*AOR* Adjusted Odds Ratios

Bold indicates significance *p* value ≤ 0.05

### Odds for no radiation therapy

When adjusted for ER/PR status alone (Table 2), API–rural women (AOR: 2.16, 95% CI 1.94–2.39), API–urban women (AOR: 1.33, 95% CI 1.30–1.36), Hispanic–rural women (AOR: 1.33, 95% CI 1.21–1.41), Hispanic–urban women (AOR: 1.21, 95% CI 1.19–1.23), NH-black–rural women (AOR: 1.31, 95% CI 1.24–1.38), NH-black–urban women (AOR: 1.16, 95% CI 1.14–1.18), and NH-white–rural women (AOR: 1.27, 95% CI 1.24–1.29) had an increased odds of no radiation treatment, when compared to NH-white–urban women. However, when only accounting for county-level SES, API–rural women (AOR: 2.09, 95% CI 1.89–2.32), API–urban women (AOR: 1.32, 95% CI 1.20–1.35), Hispanic–rural women (AOR: 1.27, 95% CI 1.17–1.38), Hispanic–urban women (AOR: 1.20, 95% CI 1.18–1.22), NH-black–rural women (AOR: 1.25, 95% CI 1.18–1.32), NH-black–urban women (AOR: 1.32, 95% CI 1.20–1.35), and NH-white–rural women (AOR: 1.24, 95% CI 1.21–1.26) had an increased odds of no radiation treatment, when compared to NH-white–urban women. Results in fully adjusted models reflected similar trends as ER/PR only adjusted model.

### Odds for no chemotherapy

When adjusted for ER/PR status alone (Table 2), Hispanic–rural women (AOR: 0.78, 95% CI 0.71–0.85), Hispanic–urban women (AOR: 0.83, 95% CI 0.81–0.85), NH-black–rural women (AOR: 0.82, 95% CI 0.77–0.87), NH-black–urban women (AOR: 0.82, 95% CI 0.81–0.84), NH-white–rural women (AOR: 0.93, 95% CI 0.92–0.95), API–urban women (AOR: 0.95, 95% CI 0.93–0.97), and API–rural women (AOR: 0.98, 95% CI 0.87–1.10) had an reduced odds of not receiving chemotherapy, when compared to NH-white–urban women. However, when only accounting for county-level SES, Hispanic–rural women (AOR: 0.79, 95% CI 0.72–0.82), Hispanic–urban women (AOR: 0.81, 95% CI 0.79–0.82), NH-black–rural women (AOR: 0.75, 95% CI 0.71–0.80), NH-black–urban women (AOR: 0.70, 95% CI 0.69–0.72), NH-white–rural women (AOR: 0.97, 95% CI 0.95–0.99), and API–urban women (AOR: 0.93, 95% CI 0.91–0.95) had reduced odds of not receiving chemotherapy, when compared to NH-white–urban women. Results in fully adjusted models reflected similar trends as ER/PR only adjusted model.

### Odds for late-stage diagnosis

When adjusted for ER/PR status alone (Table 3), NH-black–rural women (AOR: 1.37, 95% CI 1.30–1.45), NH-black–urban women (AOR: 1.33, 95% CI 1.31– 1.35), Hispanic–urban women (AOR: 1.26, 95% CI 1.24–1.29), Hispanic–rural women (AOR 1.21, 95% CI 1.11–1.31), and NH-white–rural women (AOR 1.04, 95% CI 1.02–1.06) had an increased odds of late-stage diagnosis, when compared to NH-white–urban women. However, when only accounting for county-level SES, NH-black–rural women (AOR 1.33, 95% CI 1.26–1.41), NH-black–urban women (AOR 1.35, 95% CI 1.33–1.37), Hispanic–urban women (AOR 1.26, 95% CI 1.24–1.28), and Hispanic–rural women (AOR 1.18, 95% CI 1.09–1.28) had an increased odds of late-stage diagnosis, when compared to NH-white–urban women. Results in fully adjusted models reflected similar trends as ER/PR only adjusted model.

**Table 3:** Multivariable Adjusted Odds Ratios (AOR) for Late-Stage Diagnosis and Multivariable Hazard Ratios (AHR) for Breast Cancer Mortality, SEER women diagnosed between 2000 and 2016

		ER/PR status (95% CI)^**a**^	SES (95% CI)^b^	HCA (95% CI)^c^	Fully adjusted (95% CI)^**d**^^,^^**e**^
Odds for late-stage diagnosis					
Race/Ethnicity-Rurality					
NH-white–Urban (Referent)		1.00	1.00	1.00	1.00
NH-black–Urban		1.33 (1.31–1.35)	1.35 (1.33–1.37)	1.37 (1.34 -1.39)	1.32 (1.29–1.34)
API–Urban		1.01 (0.99–1.03)	1.01 (0.99–1.03)	1.02 (1.00–1.04)	1.01 (0.99–1.03)
Hispanic–Urban		1.26 (1.24–1.29)	1.26 (1.24–1.28)	1.27 (1.24–1.29)	1.25 (1.23–1.27)
NH-white–Rural		1.04 (1.02–1.06)	1.02 (0.99–1.04)	1.01 (0.99–1.03)	1.00 (0.98–1.02)
NH-black–Rural		1.37 (1.30–1.45)	1.33 (1.26–1.41)	1.36 (1.28–1.44)	1.29 (1.22–1.36)
API–Rural		1.02 (0.91–1.14)	0.99 (0.88–1.10)	0.97 (0.87–1.09)	0.97 (0.86–1.08)
Hispanic–Rural		1.21 (1.11–1.31)	1.18 (1.09–1.28)	1.18 (1.09–1.28)	1.17 (1.08–1.27)
Risk of breast cancer death					
Race/Ethnicity-Rurality	No. breast cancer deaths (%)^f^				
NH-white–Urban (Referent)	37,006 (8.0)	1.00	1.00	1.00	1.00
NH-black–Urban	9587 (13.6)	1.75 (1.71–1.79)	1.91 (1.87–1.96)	1.99 (1.94–2.03)	1.46 (1.43–1.50)
API–Urban	3573 (6.3)	0.89 (0.86–0.92)	0.90 (0.87–0.94)	0.91 (0.88–0.94)	0.87 (0.84–0.91)
Hispanic–Urban	6437 (8.7)	1.26 (1.23–1.30)	1.27 (1.23–1.30)	1.30 (1.27–1.33)	1.10 (1.07–1.13)
NH-white–Rural	5880 (9.2)	1.15 (1.12–1.18)	1.08 (1.05–1.12)	1.09 (1.06–1.12)	1.03 (1.00–1.07)
NH-black–Rural	849 (15.0)	1.98 (1.85–2.11)	1.89 (1.76–2.03)	1.99 (1.86–2.14)	1.42 (1.32–1.53)
API–Rural	104 (6.3)	0.84 (0.70–1.02)	0.83 (0.68–1.00)	0.79 (0.65–0.96)	0.81 (0.67- 0.98)
Hispanic–Rural	252 (9.4)	1.31 (1.16–1.48)	1.21 (1.07–1.37)	1.18 (1.04–1.34)	1.00 (0.88–1.14)

^a^Adjusted for age, SEER registry, and ER/PR status

^b^Adjusted for age, SEER registry, and county-level SES

^c^Adjusted for age, SEER registry, and county-level HCA

^d^Odds of late-stage diagnosis adjusted for age, SEER registry, ER/PR status, county-level SES, and county-level HCA

^e^Risk of breast cancer models adjusted for age, SEER registry, ER/PR status, county-level SES, county-level HCA, surgical treatment, radiation therapy, chemotherapy, and late-stage diagnosis

^f^Percentage represents the relative frequency of breast cancer death given race/ethnicity-rurality strata

*AOR* Adjusted Odds Ratios

*AHR* Adjusted Hazard Ratios

Bold indicates significance *p* value ≤ 0.05

### Risk for breast cancer death

**Fig. 1:**
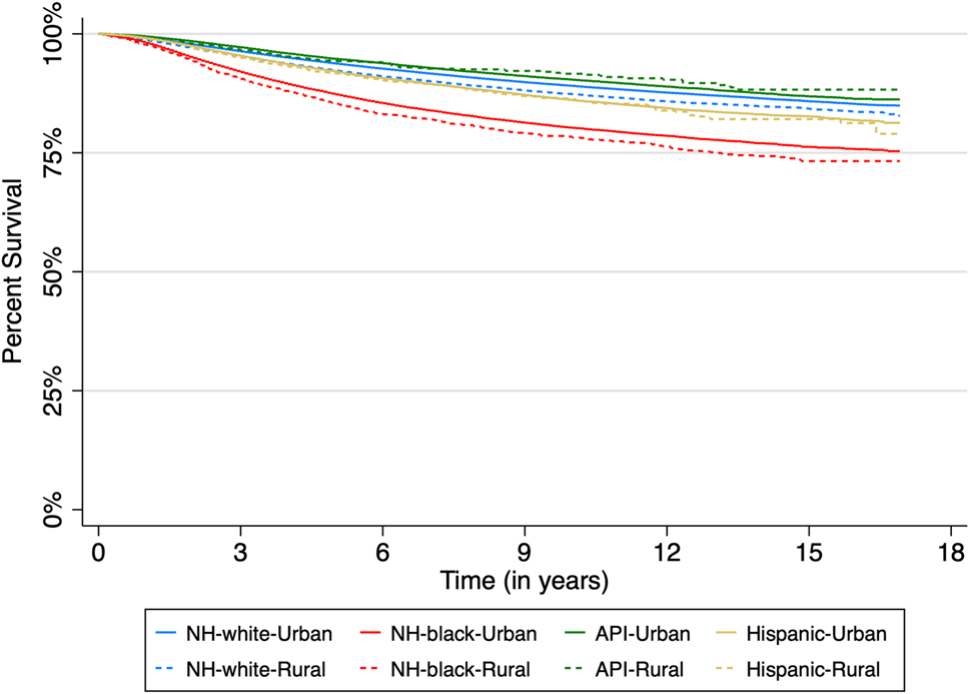
Kaplan–Meier survival plots for time-to-breast cancer death by race/ethnicity and rurality

NH-black–rural, NH-black–urban, Hispanic–rural, and Hispanic–urban women had the largest relative frequency of breast cancer death (Table 3 Fig. 1). When adjusted for ER/PR status alone, NH-black–rural women (AOR 1.98, 95% CI 1.85–2.11), NH-black–urban women (AOR 1.75, 95% CI 1.71– 1.79), Hispanic–rural women (AOR 1.31, 95% CI 1.16–1.48), Hispanic–urban women (AOR 1.26, 95% CI 1.23–1.30), and NH-white–rural women (AOR 1.15, 95% CI 1.12–1.18) had an increased risk of breast cancer death, when compared to NH-white–urban women. However, when only accounting for county-level SES, NH-black–rural women (AOR 1.89, 95% CI 1.76–2.03), NH-black–urban women (AOR 1.91, 95% CI 1.87– 1.96), Hispanic–rural women (AOR 1.21, 95% CI 1.07–1.37), Hispanic–urban women (AOR 1.27, 95% CI 1.23–1.30), and NH-white–rural women (AOR 1.08, 95% CI 1.05–1.12) had an increased risk of breast cancer death, when compared to NH-white–urban women. Results in fully adjusted models, accounting for ER/PR status, county-level SES, and county-level HCA revealed NH-black–rural women (AOR 1.42, 95% CI 1.32–1.53), NH-black–urban women (AOR 1.46, 95% CI 1.43–1.50), Hispanic–urban women (AOR 1.10, 95% CI 1.07–1.13), and NH-white–rural women (AOR 1.03, 95% CI 1.00–1.07) had an increased risk of breast cancer death, when compared to NH-white–urban women. We further performed analyses examining the interaction between race and rurality on breast cancer outcomes stratified by ER/PR status (Supplemental Tables 1, 2) and observed similar trends. Additionally, we assessed the multiplicative and additive interaction between race/ethnicity and rurality (Supplemental Tables 3, 4).

## Discussion

The study sought to examine the intersectionality of rurality and race/ethnicity on breast cancer outcomes. Overall, we observed increased odds for late-stage breast cancer diagnosis, odds of no receipt of breast cancer treatments (surgical, radiation therapy, and chemotherapy), and increased risk of breast cancer death among NH-black and Hispanic women, regardless of rurality, when compared with NH-white–urban women. Rural and urban API women generally had better breast cancer outcomes, with a 10–20% reduced risk of breast cancer death when compared with NH-white–urban women. NH-black and Hispanic women were less likely to receive treatment, and in turn, were at higher risks for later stage disease and breast cancer mortality. Further, when examining possible confounders, results in fully adjusted models mirrored trends in ER/PR only adjusted models for late-stage disease, odds of no chemotherapy, radiation, or surgical treatment.

Prior studies have observed associations between race/ethnicity or rural/urban status with both breast cancer outcomes and mortality [28, 29, 40]. Some studies have explored the rural–urban differences in breast cancer incidence [41], survival [42], mortality [28, 29, 43], and focused on specific regions, while the results of our current study aimed to illuminate a nationally representative relationship between race and rurality with several breast cancer outcomes. For instance, *Moore *et al. *(2018)* observed that specifically for NH-black and Hispanic women, geographic areas of high breast cancer mortality or ‘hot spots’ were prevalent throughout the southeastern U.S. for NH-black women, and among Hispanic women within the southwest region of the U.S. [40]. Further, these hot spot areas were characterized by having poorer social determinants of health factors including lower educational attainment and lower household income; however, NH-black and Hispanic breast cancer mortality hot spots were not characterized by greater rurality [40]. This finding is concordant with our current study as we observed that regardless of NH-black and Hispanic women living in rural or urban communities, they had poorer breast cancer outcomes. In contrast to the study design of *Moore *et al. *(2018)* which analyzed mortality data aggregated to the county level, our current study provides more contextual evidence on the possible association between race and place with breast cancer outcomes while using patient-level data with more granular information regarding breast cancer status, treatments, and survival.

### Odds of no treatment surgery, chemotherapy, radiation

NH-black–urban and NH-black–rural women were nearly twice as likely to not receive surgical treatment when compared to NH-white–urban women. Most racial/ethnic minority women of rural and urban communities were more likely to receive chemotherapy; however, these racial minorities had increased odds of not receiving radiation therapy or surgical treatment. Chemotherapy treatment is more common among women with late-stage diagnosis compared with women with early-stage diagnosis, 68% vs. 27% respectively. NH-black and Hispanic women are 30% more likely to be diagnosed with late-stage breast cancer compared to NH-white women, therefore they are more likely to receive chemotherapy. Other studies suggest that black women are more likely to have delays in their follow-up care for breast cancer when compared with white women. As suggested by *Babatunde *et al*. (2021)*, black women were more likely to receive surgery 8 days later, chemotherapy 7 days later, radiation therapy 3 days later, and adjuvant hormone therapy 28 days later than their white counterparts [44].

The pattern for odds ratios by ER/PR breast cancer status was similar to that for odds of no surgical treatment, with NH-black–urban and rural women having the highest odds of receiving no surgery across all breast cancer status. The most substantial differences exist for ER+/PR+ status followed by ER+ /PR– or ER–/PR+ status—indicating that lack of surgical treatment may also be a major factor for disparities in risk for breast cancer death. Yet, in fully adjusted models, NH-black–urban women are still twice as likely—with NH-black–rural women close behind—to not receive surgery compared to NH-white–urban women. It is plausible that when even accounting for socioeconomic barriers and health insurance, NH-black and Hispanic women still face additional barriers of racial micro- and macro-aggressions, lack of representation within healthcare systems, and mistrust of health professionals that in turn reduce their likelihood of receiving timely and appropriate cancer care [45–48]. Several themes have emerged as a result of investigation into the cause of racial/ethnic disparities in breast cancer treatment, including persistent medical mistrust by black women [49, 50] and structural and interpersonal biases in cancer care [51]. Studies have also indicated that even when surgery is available to black women, existing comorbidities are major barriers to positive outcomes following surgery [52].

### Late-stage diagnosis

Consistent with literature regarding breast cancer disparities [53], NH–black women and Hispanic women had the highest odds for late-stage diagnosis among all racial/ethnic groups in fully adjusted models. Further *Warner *et al*. (2012)* observed that black and Hispanic women experienced longer delays and time to diagnoses compared with non-Hispanic white women [54]. Among all groups, NH–black urban and rural women had higher rates of late-stage disease. Our findings are similar to those of *Mobley *et al*. (2021)* using United States Cancer Statistic (USCS) data, where they reported persistent hot spots over a 10-year period for late-stage breast cancer among poor, rural, African American, and Hispanic communities, but not in poor, rural, and White communities [27]. Likewise, we also observed NH-black and Hispanic women had from a 17% to 32% increased odds of late-stage breast cancer diagnosis, regardless of rurality, when compared to NH-white women. Additionally, *Williams *et al*. (2016)* reported that among a cohort of 29,410 Missouri rural and urban women, black women had a 50% increased odds of late-stage breast cancer diagnosis, compared to white women, and non-metropolitan or rural counties had 11% increased odds of late-stage diagnosis, when compared to their urban counterparts [55]. In the present study, NH-black women were nearly twice as likely as other racial/ethnic groups to be diagnosed with ER-/PR-status. These findings are consistent with existing literature outlining racial/ethnic disparities in breast cancer type and stage [56–61].

### Risk of breast cancer death

In fully adjusted models, NH–black–urban/rural women and Hispanic–urban women had the highest risk of breast cancer death—consistent with trends observed for the last 40 years [62]. Although the risk for breast cancer death remains substantially higher for NH-black women compared to NH-white–urban women, the differences in risk breast cancer mortality stratified by HR cancer status may give insight on possible drivers of these disparities. When observing odds for late-stage disease, type of treatment, and risk of breast cancer death by ER/PR status, racial/ethnic and urban–rural differences were the smallest for ER-/PR- status. This may be explained largely by women with triple-negative breast cancer (TNBC) who fall into this category [63, 64]. The lack of available treatments for TNBC means that generally no group—regardless of geographical location or race/ethnicity——can benefit over another from targeted therapies for this subtype [65, 66]. Interestingly, larger disparities were seen among women with ER+ and/or PR+ status, for which targeted therapies are currently available, as odds for risk of breast cancer death for both urban and rural NH-black women compared to other groups were considerably higher.

It is well documented that the large gap in breast cancer survival between black and white women began in the 1980s, following the introduction of adjuvant endocrine therapy for ER+ breast cancer status and continued with subsequent therapies for PR+ and HER2+ subtypes[67]. While much discussion has focused on the prevalence of hormone receptor negative cancers (particularly TNBC) among black women as a major driver of racial/ethnic disparities in breast cancer survival [58, 68], it is evident that there are additional barriers to survival for black women with ER+ or PR+ disease despite availability of targeted therapy. In fact, a cohort study of Chicago women observed a fourfold increased risk of ER+ /PR+ breast cancer among black women, when compared to white women [69]. While it has been suggested that racial differences in survival may be a result of decreased response to treatment due to molecular differences in ER+ /PR+ tumors of black women [69], research has shown that even when available, black women are less likely to adhere to adjuvant endocrine therapy compared to other racial/ethnic groups [70, 71]. Adherence may in part be due to black women reporting greater burden of side effects, differential risk perceptions, and lack of shared treatment decision making [72].

### Strengths and limitations

This study should be considered with respect to several strengths and limitations. First, SEER 18 is not a comprehensive cancer surveillance and does not have information regarding every cancer breast cancer diagnosis within the U.S. However, SEER covers approximately 28% of U.S. population among 18 geographic areas and cancer registries; therefore, the results of these data serve as the closest approximation to the U.S. general cancer patient population. Moreover, SEER sampling allows for exploration between race/ethnicity and rurality with breast cancer outcomes with large patient sample. For geographic location, SEER utilizes the county Federal Information Processing Standard (FIPS) code at the time of each patient’s diagnosis, and we are unable to discern variability in a patients’ residence over their life course. With this respect, our use of county-level SES and HCA factors is limited by ecologic or aggregation biases, and thus may not be reflective of both more granular neighborhood measures such as census block or tract level data (these data are not available for linkages with SEER patient data). Further, the built and social environment may not be static, and the interpretations of county-level factors influence on these analyses should be tempered with this respect. Nevertheless, this study was inclusive of eight race/ethnicity and geographic intersectional identities and may provide a more granular understanding on the effects of race and place on breast cancer outcomes.

### Conclusion

Of particular interest, we observed consistently higher odds for late-stage breast cancer diagnosis and no receipt of breast cancer surgery, and higher risk for breast cancer death among NH-black–urban, NH-black–rural, Hispanic–urban, and Hispanic–rural women compared to NH-white–urban women in fully adjusted models. Our findings are consistent with a few studies that have observed worse outcomes among rural breast cancer women compared to urban. These studies cite individual- and community-level psychosocial factors as possible drivers of the observed differences [73]. Given the marked disparities in breast cancer outcomes for both NH-black and Hispanic women, regardless of rurality, future studies and public health initiatives should consider strategies and programming that are culturally tailored and inclusive for racial/ethnic minorities. Further, increasing availability of genetic testing, screening resources/services, and reducing economic and interpersonal barriers to follow-up care after first primary diagnosis may help considerably in reducing the given breast cancer health inequities for NH-black and Hispanic women living in both rural and urban communities.

## Supplementary Information

Below is the link to the electronic supplementary material.

Supplementary file1 (DOCX 17 KB)

Supplementary file2 (DOCX 16 KB)

Supplementary file3 (DOCX 18 KB)

Supplementary file4 (DOCX 16 KB)

## Data Availability

The datasets generated and/or analyzed during the current study are available in the Surveillance Epidemiology and End Results (SEER) 18 registrations custom data (with additional treatment fields repository, (http://seer.cancer.gov).

## Abbreviations

SEER: Surveillance Epidemiology and End Results
AORs: Adjusted odds ratios
AHRs: Adjusted hazard ratios
CIs: 95% Confidence intervals
RUCC: Rural–Urban Continuum Codes
NH-white: Non-Hispanic white
NH-black: Non-Hispanic black
API: Asian/Pacific Islander
U.S.: United States
SES: Socioeconomic/demographic
HCA: Health care access
ACS: American Community Survey
ER: Estrogen receptor
PR: Progesterone receptor
TNBC: Triple-negative breast cancer
FIPS: Federal Information Processing Standard
