# Supplementary material for: Exploring the intersectionality of race/ethnicity with rurality on breast cancer outcomes: SEER analysis, 2000–2016
Source: Breast Cancer Res Treat. 2022 Dec 15;197(3):633–45. doi: 10.1007/s10549-022-06830-x (PMC9883364; doi:10.1007/s10549-022-06830-x)
Supplement: Supplementary file 1 — Supplementary file1 (DOCX 17 KB) [file 10549_2022_6830_MOESM1_ESM.docx]

| Supplemental Table 1: Multivariable Adjusted Odds Ratios for No Surgical Treatment, No Chemotherapy, and No Radiation Therapy for SEER Breast Cancer women diagnosed between 2000 through 2016, stratified by ER/PR status. | | | |
| --- | --- | --- | --- |
|  | **ER+/PR+ Status**  **AOR (95% CI)** ^a^ | **ER+/PR- or ER-/PR+ Status**  **AOR (95% CI)** ^a^ | **ER-/PR- Status**  **AOR (95% CI)** ^a^ |
| Odds for No Surgical Treatment | | | |
| Race/ Ethnicity-Rurality |  |  |  |
| NH-white – Urban (Referent) | 1.00 | 1.00 | 1.00 |
| NH-black – Urban | 2.15 (2.04 – 2.27) | 2.05 (1.87 – 2.26) | 1.74 (1.62 – 1.88) |
| API – Urban | 1.45 (1.39 – 1.58) | 1.36 (1.20 – 1.55) | 1.35 (1.21 – 1.50) |
| Hispanic – Urban | 1.62 (1.53 – 1.71) | 1.58 (1.42 – 1.75) | 1.58 (1.45 – 1.72) |
| NH-white – Rural | 1.03 (0.96 – 1.11) | 1.01 (0.87 – 1.17) | 0.97 (0.85 – 1.12) |
| NH-black – Rural | 1.74 (1.44 – 1.58) | 1.68 (1.20 – 2.36) | 1.54 (1.19 – 1.98) |
| API – Rural | 0.70 (0.41 – 1.20) | 1.23 (0.56 – 2.69) | 1.39 (0.67 – 2.88) |
| Hispanic – Rural | 1.48 (1.18 – 1.86) | 1.15 (0.66 – 2.01) | 1.52 (1.00 – 2.31) |
| Odds for No Radiation Therapy | | | |
| Race/ Ethnicity-Rurality |  |  |  |
| NH-white – Urban (Referent) | 1.00 | 1.00 | 1.00 |
| NH-black – Urban | 1.20 (1.17 – 1.23) | 1.17 (1.12 – 1.22) | 1.01 (0.97 – 1.04) |
| API – Urban | 1.30 (1.27 – 1.33) | 1.32 (1.26 – 1.40) | 1.36 (1.29 – 1.42) |
| Hispanic – Urban | 1.19 (1.17 – 1.22) | 1.19 (1.13 – 1.24) | 1.16 (1.12 – 1.21) |
| NH-white – Rural | 1.19 (1.16 – 1.22) | 1.27 (1.20 – 1.34) | 1.16 (1.10 – 1.22) |
| NH-black – Rural | 1.22 (1.13 – 1.32) | 1.25 (1.07 – 1.46) | 1.08 (0.97 – 1.21) |
| API – Rural | 2.14 (1.89 – 2.43) | 1.72 (1.29 – 2.30) | 2.08 (1.59 – 2.71) |
| Hispanic – Rural | 1.26 (1.14 – 1.40) | 1.42 (1.12 – 1.79) | 1.00 (0.83 – 1.22) |
| Odds for No Chemotherapy | | | |
| Race/ Ethnicity-Rurality |  |  |  |
| NH-white – Urban (Referent) | 1.00 | 1.00 | 1.00 |
| NH-black – Urban | 0.81 (0.79 – 0.83) | 0.82 (0.78 – 0.86) | 0.88 (0.85 – 0.92) |
| API – Urban | 0.95 (0.92 – 0.97) | 0.90 (0.85 – 0.95) | 1.01 (0.96 – 1.07) |
| Hispanic – Urban | 0.83 (0.81 – 0.85) | 0.76 (0.72 – 1.40) | 0.92 (0.87 – 0.96) |
| NH-white – Rural | 0.94 (0.92 – 0.97) | 0.96 (0.91 – 1.03) | 1.06 (1.00 -1.12) |
| NH-black – Rural | 0.79 (0.73 – 0.87) | 0.81 (0.68 – 0.96) | 0.98 (0.86 – 1.12) |
| API – Rural | 1.00 (0.86 – 1.15) | 1.02 (0.74 – 1.40) | 1.17 (0.87 – 1.57) |
| Hispanic – Rural | 0.81 (0.72 – 0.90) | 0.93 (0.72 – 1.21) | 0.76 (0.60 – 0.97) |
| ^a^ Adjusted for age, SEER registry, county-level SES, and county-level HCA.  AOR = Adjusted Odds Ratios.  Bold indicates significance *p* value ≤ 0.05. | | | |
