# Supplementary material for: Exploring the intersectionality of race/ethnicity with rurality on breast cancer outcomes: SEER analysis, 2000–2016
Source: Breast Cancer Res Treat. 2022 Dec 15;197(3):633–45. doi: 10.1007/s10549-022-06830-x (PMC9883364; doi:10.1007/s10549-022-06830-x)
Supplement: Supplementary file 2 — Supplementary file2 (DOCX 16 KB) [file 10549_2022_6830_MOESM2_ESM.docx]

| Supplemental Table 2: Multivariable Adjusted Odds Ratios (AOR) for Late-Stage Diagnosis and Multivariable Hazard Ratios (AHR) for Breast Cancer Mortality, SEER women diagnosed between 2000 through 2016, stratified by ER/PR status. | | | |
| --- | --- | --- | --- |
|  | **ER+/PR+ Status**  **(95% CI)** ^a, b^ | **ER+/PR- or ER-/PR+ Status**  **(95% CI)** ^a, b^ | **ER-/PR- Status**  **(95% CI)** ^a, b^ |
| Odds for Late-Stage Diagnosis | | | |
| Race/ Ethnicity-Rurality |  |  |  |
| NH-white – Urban (Referent) | 1.00 | 1.00 | 1.00 |
| NH-black – Urban | 1.34 (1.30 – 1.37) | 1.29 (1.23 – 1.35) | 1.29 (1.25 – 1.34) |
| API – Urban | 1.01 (0.98 – 1.03) | 0.99 (0.93 – 1.04) | 1.05 (1.00 – 1.10) |
| Hispanic – Urban | 1.26 (1.24 – 1.29) | 1.29 (1.23 – 1.35) | 1.23 (1.18 – 1.28) |
| NH-white – Rural | 1.02 (0.99 – 1.05) | 0.96 (0.90 – 1.01) | 1.01 (0.96 – 1.06) |
| NH-black – Rural | 1.22 (1.13 – 1.33) | 1.28 (1.10 – 1.50) | 1.37 (1.23 – 1.53) |
| API – Rural | 0.90 (0.78 – 1.03) | 1.05 (0.78 – 1.41) | 1.17 (0.90 – 1.53) |
| Hispanic – Rural | 1.12 (1.01 – 1.25) | 1.22 (0.96 – 1.55) | 1.22 (1.01 – 1.48) |
| Risk of Breast Cancer Death | | | |
| Race/ Ethnicity-Rurality |  |  |  |
| NH-white – Urban (Referent) | 1.00 | 1.00 | 1.00 |
| NH-black – Urban | 1.57 (1.51 – 1.64) | 1.51 (1.43 – 1.60) | 1.32 (1.27 – 1.38) |
| API – Urban | 0.92 (0.87 – 0.97) | 0.93 (0.85 – 1.01) | 0.78 (0.74 – 0.84) |
| Hispanic – Urban | 1.10 (1.05 – 1.14) | 1.16 (1.08 – 1.24) | 1.05 (1.00 – 1.10) |
| NH-white – Rural | 1.04 (0.99 – 1.09) | 1.08 (1.00 – 1.16) | 1.06 (1.00 – 1.13) |
| NH-black – Rural | 1.50 (1.31 – 1.70) | 1.33 (1.10 – 1.61) | 1.29 (1.15 – 1.46) |
| API – Rural | 0.85 (0.64 – 1.13) | 0.62 (0.37 – 1.05) | 0.76 (0.54 – 1.09) |
| Hispanic – Rural | 1.06 (0.87 – 1.29) | 1.00 (0.72 – 1.38) | 0.95 (0.75 – 1.21) |
| ^a^Odds of late-stage diagnosis models adjusted for age, SEER registry, county-level SES, and county-level HCA.  ^b^Risk of breast cancer models adjusted for age, SEER registry, ER/PR status, county-level SES, county-level HCA, surgical treatment, radiation therapy, chemotherapy, and late-stage diagnosis.  AOR = Adjusted Odds Ratios.  AHR = Adjusted Hazard Ratios.  Bold indicates significance *p* value ≤ 0.05. | | | |
