# Supplementary material for: Exploring the intersectionality of race/ethnicity with rurality on breast cancer outcomes: SEER analysis, 2000–2016
Source: Breast Cancer Res Treat. 2022 Dec 15;197(3):633–45. doi: 10.1007/s10549-022-06830-x (PMC9883364; doi:10.1007/s10549-022-06830-x)
Supplement: Supplementary file 3 — Supplementary file3 (DOCX 18 KB) [file 10549_2022_6830_MOESM3_ESM.docx]

| Supplemental Table 3: Multivariable Association of Rural/Urban Status Presented as Adjusted Odds Ratios (AOR) for No Surgical Treatment, No Chemotherapy, and No Radiation Therapy for SEER Breast Cancer women diagnosed between 2000 through 2016, stratified by race/ethnicity. | | | | |
| --- | --- | --- | --- | --- |
|  | **ER/PR Status**  **AOR (95% CI)** ^a^ | **SES**  **AOR (95% CI) ^b^** | **HCA**  **AOR (95% CI) ^c^** | **Fully Adjusted**  **AOR (95% CI)** ^d^ |
| Odds for No Surgical Treatment | | | | |
| Among NH-White |  |  |  |  |
| Urban (Referent) | 1.00 | 1.00 | 1.00 | 1.00 |
| Rural | 1.01 (0.96 – 1.06) | 1.06 (1.01 – 1.12) | 1.08 (1.03 – 1.14) | 1.03 (0.97 – 1.09) |
| Among NH-Black |  |  |  |  |
| Urban (Referent) | 1.00 | 1.00 | 1.00 | 1.00 |
| Rural | 0.91 (0.80 – 1.03) | 0.91 (0.80 – 1.05) | 0.95 (0.83 – 1.08) | 0.89 (0.77 – 1.02) |
| Among API |  |  |  |  |
| Urban (Referent) | 1.00 | 1.00 | 1.00 | 1.00 |
| Rural | 0.67 (0.48 – 0.94) | 0.60 (0.42 – 0.86) | 0.72 (0.50 – 1.02) | 0.59 (0.41 – 0.86) |
| Among Hispanic |  |  |  |  |
| Urban (Referent) | 1.00 | 1.00 | 1.00 | 1.00 |
| Rural | 0.97 (0.81 – 1.17) | 1.02 (0.84 – 1.22) | 1.09 (0.90 – 1.31) | 1.04 (0.85 – 1.26) |
| Odds for No Radiation Therapy | | | | |
| Among NH-White |  |  |  |  |
| Urban (Referent) | 1.00 | 1.00 | 1.00 | 1.00 |
| Rural | 1.23 (1.21 – 1.26) | 1.21 (1.18 – 1.23) | 1.22 (1.20 – 1.25) | 1.18 (1.16 – 1.21) |
| Among NH-Black |  |  |  |  |
| Urban (Referent) | 1.00 | 1.00 | 1.00 | 1.00 |
| Rural | 1.20 (1.13 – 1.28) | 1.04 (0.97 – 1.11) | 1.16 (1.09 – 1.24) | 1.02 (0.95 – 1.09) |
| Among API |  |  |  |  |
| Urban (Referent) | 1.00 | 1.00 | 1.00 | 1.00 |
| Rural | 1.91 (1.71 – 2.12) | 1.73 (1.54 – 1.94) | 1.85 (1.64 – 2.09) | 1.67 (1.47 – 1.90) |
| Among Hispanic |  |  |  |  |
| Urban (Referent) | 1.00 | 1.00 | 1.00 | 1.00 |
| Rural | 1.23 (1.13 – 1.34) | 1.23 (1.13 – 1.34) | 1.24 (1.13 – 1.35) | 1.23 (1.12 – 1.35) |
| Odds for No Chemotherapy | | | | |
| Among NH-White |  |  |  |  |
| Urban (Referent) | 1.00 | 1.00 | 1.00 | 1.00 |
| Rural | 0.93 (0.91 – 0.95) | 0.96 (0.94 – 0.98) | 0.96 (0.94 – 0.98) | 0.96 (0.94 – 0.98) |
| Among NH-Black |  |  |  |  |
| Urban (Referent) | 1.00 | 1.00 | 1.00 | 1.00 |
| Rural | 1.01 (0.95 – 1.08) | 1.03 (0.96 – 1.11) | 1.04 (0.97 – 1.11) | 1.02 (0.94 – 1.10) |
| Among API |  |  |  |  |
| Urban (Referent) | 1.00 | 1.00 | 1.00 | 1.00 |
| Rural | 1.06 (0.95 – 1.19) | 1.16 (1.02 – 1.31) | 1.22 (1.07 – 1.38) | 1.26 (1.10 – 1.45) |
| Among Hispanic |  |  |  |  |
| Urban (Referent) | 1.00 | 1.00 | 1.00 | 1.00 |
| Rural | 0.94 (0.85 – 1.03) | 1.01 (0.92 – 1.11) | 1.05 (0.96 – 1.16) | 0.99 (0.89 – 1.09) |
| ^a^Adjusted for age, SEER registry, and ER/PR status.  ^b^Adjusted for age, SEER registry, and county-level SES.  ^c^Adjusted for age, SEER registry, and county-level HCA.  ^d^Adjusted for age, SEER registry, ER/PR status, county-level SES, and county-level HCA.  AOR = Adjusted Odds Ratios.  Bold indicates significance *p* value ≤ 0.05. | | | | |
