# Supplementary material for: Exploring the intersectionality of race/ethnicity with rurality on breast cancer outcomes: SEER analysis, 2000–2016
Source: Breast Cancer Res Treat. 2022 Dec 15;197(3):633–45. doi: 10.1007/s10549-022-06830-x (PMC9883364; doi:10.1007/s10549-022-06830-x)
Supplement: Supplementary file 4 — Supplementary file4 (DOCX 16 KB) [file 10549_2022_6830_MOESM4_ESM.docx]

| Supplemental Table 4: Multivariable Association of Rural/Urban Status with Late Stage Diagnosis, Presented as Adjusted Odds Ratios (AOR), and Breast Cancer Death, Presented as Hazard Ratios (AHR), among SEER women diagnosed between 2000 through 2016, stratified by race/ethnicity. | | | | |
| --- | --- | --- | --- | --- |
|  | **ER/PR Status**  **(95% CI)** ^a^ | **SES**  **(95% CI) ^b^** | **HCA**  **(95% CI) ^c^** | **Fully Adjusted**  **(95% CI)** ^d, e^ |
| Odds for Late-Stage Diagnosis | | | | |
| Among NH-White |  |  |  |  |
| Urban (Referent) | 1.00 | 1.00 | 1.00 | 1.00 |
| Rural | 1.04 (1.02 – 1.06) | 1.01 (0.99 – 1.03) | 1.01 (0.99 – 1.03) | 1.00 (0.98 – 1.03) |
| Among NH-Black |  |  |  |  |
| Urban (Referent) | 1.00 | 1.00 | 1.00 | 1.00 |
| Rural | 1.03 (0.97 – 1.10) | 1.02 (0.95 – 1.08) | 0.99 (0.93 – 1.06) | 0.99 (0.93 – 1.06) |
| Among API |  |  |  |  |
| Urban (Referent) | 1.00 | 1.00 | 1.00 | 1.00 |
| Rural | 1.01 (0.91 – 1.14) | 0.91 (0.81 – 1.03) | 0.92 (0.81 – 1.04) | 0.89 (0.78 – 1.01) |
| Among Hispanic |  |  |  |  |
| Urban (Referent) | 1.00 | 1.00 | 1.00 | 1.00 |
| Rural | 0.97 (0.89 – 1.06) | 0.94 (0.86 – 1.03) | 0.92 (0.84 – 1.01) | 0.93 (0.85 – 1.02) |
| Risk of Breast Cancer Death | | | | |
| Among NH-White |  |  |  |  |
| Urban (Referent) | 1.00 | 1.00 | 1.00 | 1.00 |
| Rural | 1.14 (1.11 – 1.17) | 1.07 (1.03 – 1.10) | 1.08 (1.05 – 1.12) | 1.02 (0.99 – 1.06) |
| Among NH-Black |  |  |  |  |
| Urban (Referent) | 1.00 | 1.00 | 1.00 | 1.00 |
| Rural | 1.12 (1.05 – 1.20) | 1.04 (0.96 – 1.13) | 1.08 (1.00 – 1.17) | 1.02 (0.94 – 1.11) |
| Among API |  |  |  |  |
| Urban (Referent) | 1.00 | 1.00 | 1.00 | 1.00 |
| Rural | 0.98 (0.80 – 1.19) | 0.99 (0.79 – 1.22) | 0.89 (0.72 – 1.11) | 0.93 (0.73 – 1.18) |
| Among Hispanic |  |  |  |  |
| Urban (Referent) | 1.00 | 1.00 | 1.00 | 1.00 |
| Rural | 1.05 (0.93 – 1.19) | 0.94 (0.82 – 1.08) | 0.83 (0.72 – 0.95) | 0.89 (0.77 – 1.03) |
| ^a^Adjusted for age, SEER registry, and ER/PR status.  ^b^Adjusted for age, SEER registry, and county-level SES.  ^c^Adjusted for age, SEER registry, and county-level HCA.  ^d^Odds of late-stage diagnosis adjusted for age, SEER registry, ER/PR status, county-level SES, and county-level HCA.  ^e^Risk of breast cancer models adjusted for age, SEER registry, ER/PR status, county-level SES, county-level HCA, surgical treatment, radiation therapy, chemotherapy, and late-stage diagnosis.  AOR = Adjusted Odds Ratios.  AHR = Adjusted Hazard Ratios.  Bold indicates significance *p* value ≤ 0.05. | | | | |
